# Memory improving effect of silkworm larva on insulin resistance related cognitive impairment model

**DOI:** 10.1371/journal.pone.0328847

**Published:** 2025-07-28

**Authors:** Joohyun Hwang, Junhyuk Choi, Seung-Yun Cha, In-Seo Lee, Ji Hye Yoon, Duk Jin Jung, Chang Wook Lee, Seong Ryul Kim, Ji Hae Lee, Byeongyeob Jeon, Ji-Ho Park, Sungho Maeng, Hyunwoo Park

**Affiliations:** 1 Age-Tech Service Convergence Major, Graduate School of East–West Medical Science. Kyung Hee University, Yongin-si, Republic of Korea; 2 Department of East-West Medicine, Graduate School of East–West Medical Science, Kyung Hee University, Yongin-si, Republic of Korea; 3 Department of Agricultural Biology, National Institute of Agricultural Sciences, Rural Development Administration, Wanju, Republic of Korea; 4 QB Department of Agricultural Biology, National Institute of Agricultural Sciences, Rural Development Administration, Wanju, Republic of Korea; 5 QBM co., ltd., Seocho-gu, Seoul, Republic of Korea; 6 Health Park co., ltd., Seoul National University, Seoul, Republic of Korea; University of Waterloo, CANADA

## Abstract

Hongjam (HJ) is the steamed and freeze-dried powder of larva-stage silkworm (*Bombyx mori*) rich in protein, unsaturated fatty acids, and minerals. Silkworm products have traditionally been used for medical purposes, and their effectiveness in diabetic and neurodegenerative diseases has been studied. In particular, the anti-inflammatory-antioxidant, blood sugar-lowering, and neuroprotective effects are expected to be useful for the prevention and treatment of dementia, especially in a model of dementia related to insulin resistance. Most animal models of Alzheimer’s disease (AD) are based on genetic factors and research based on these models does not explain the pathophysiology of sporadic AD. Therefore, no drug can effectively delay the progression of AD. We hypothesized that HJ may improve cognitive function in an insulin resistance model which is considered one of the causes of sporadic AD. Insulin resistance was induced by a high-fat diet and streptozotocin injection. Additionally, the effect of HJ was tested in in vitro cultured hippocampal slices treated with an N-methyl-D-aspartate antagonist. At the given dose, HJ did not affect on the body weight but lowered blood glucose concentration, improved spatial memory in the Morris water maze and avoidance memory in the passive avoidance tests which was related to hippocampal brain-derived neurotrophic factor. In hippocampal slices, HJ strengthened long-term potentiation, which was suppressed by AP5. Thus, HJ improved cognitive functions in an insulin-resistance related dementia model and may be useful in treating sporadic AD.

## Introduction

Despite the socioeconomic impact of senile dementia, there is no adequate treatment [1]. Many natural and novel synthetic compounds with anti-inflammatory, antioxidant, neuroprotective, and amyloid disposal effects, which are expected to treat senile dementia are being tested in preclinical and clinical stages, yet unsatisfactory [2]. It is important to prevent the neurodegenerative process before it reaches into later stages, and early diagnosis is necessary for early preventive treatment. A better understanding of the pathophysiology in the early stages of dementia is required for early diagnosis. Currently, many factors have been revealed that precede the deposition of amyloid beta (Aβ) and hyperphosphorylated-tau [3]. Among these, failure in energy metabolism due to insulin resistance within the brain is known as one of the major initial causes of dementia [4].

Transgenic mouse Alzheimer’s disease (AD) models are widely used, and they provide good insights into the neurodegenerative process caused by Aβ protein toxicity. However, they resemble the hereditary form of AD and do not model the sporadic process of AD. Therefore, current transgenic models are inappropriate to understand the pathophysiologic process of sporadic AD and to develop early diagnostic methods of AD [5]. Conversely, rodents administered streptozotocin combined with high-fat diets are animal models with abnormal brain energy metabolism and insulin resistance which is considered an important factor causing sporadic dementia [6]. It resembles many aspects of AD, known with cognitive impairments, amyloid and tau pathology, neuroinflammation, oxidative stress, impaired insulin signaling, and neurovascular changes [5].

Silkworm is the larva of silk-producing moth, scientific name *Bombyz mori*, which has been traditionally used for many medicinal purposes such as for flatulence, spasms, and phlegm [7]. However, at the larva stage, the silk gland containing silk protein is hard to chew, so to make it edible, ‘Hongjam’ (HJ) is a steamed and freeze-dried powder of the silkworm. HJ contains more than 70% of protein and is rich in omega-3 fatty acids and essential minerals [8]. Silkworm produces many silk proteins such as fibroin and sericin [9], and these proteins effectively prevent liver damage, Parkinson’s disease, and lowering blood pressure and blood sugar [10–12]. In particular, 1-deoxynojirimycin (1-DNJ), which accumulates in the body of silkworm pupae by eating mulberry leaves, has hypoglycemic effects by α-glucosidase inhibition, activation of glycolytic enzymes, and inhibition of enzymes involved in gluconeogenesis [13]. Silkworm-derived substances are beneficial for neurological diseases. Silkworm pupae show neuroprotective effects by enhancing cholinergic function and antioxidant properties [14]. In scopolamine-treated mice, silkworm protein improves memory performance in the Morris water maze and passive avoidance test [15]. Also, a silkworm product called BF-7 improves IQ scores and the ability to concentrate in children [16]. Thus, silkworm products can potentially improve memory in dementia. Therefore, in this study, we hypothesized that HJ has a positive effect on the cognitive impairment model caused by insulin resistance in the brain. Hence, HJ was administered to *in vivo* and in vitro animal models, and its behavioral, biochemical, and electrophysiological effects were evaluated.

## Materials and methods

### Animals

The effect of HJ on cognitive impairments was tested on two cohorts of animals. The first cohort was an *in vivo* measurement on mice using behavioral and biochemical methods. The second cohort was a measurement of *in vitro* effect on rat hippocampal slices using electrophysiological methods. For sacrificing, animals were deeply anesthetized with isoflurane, then decapitated for brain extraction. All animal studies were conducted in accordance with the National Institutes of Health (NIH) Guide for the Care and Use of Laboratory Animals to minimize suffering. The protocols were approved by the Institutional Animal Care and Use Committee of Kyung Hee University (KHUASP-21-068).

### Preparation of HJ

The F1 hybrid of *Bombyx mori* Jam 311 and Jam 312 (Golden Silk strain) larvae were reared by feeding mulberry leaves [17]. Mature silkworm larvae were selected and processed as previously reported [18]. Briefly, harvested silkworms were steamed for 130 min and freeze-dried (FDT-8612, Operon Ltd, Kimpo, Korea) at −50°C for 24 h. They were cut by a multipurpose mill (DSMP-370, Duksan Co., Ltd., Siheung, Korea) and ground into particles using a roller mill (Duksan Co., Ltd.). The nutrients of the particles were extracted in methanol 80% (v/v), followed by evaporation of the solvent using a vacuum desiccator containing dried silica gels at room temperature. For experimental use, the extract was dissolved in distilled water.

### HPLC measurement of 1-DNJ

A TSKgel Amide-80 column (4.6 × 250 mm, 5 μm, Merck, Darmstadt, Germany) was used with Waters Alliance e2695 Separations Module, 2424, (Waters Corporation, Milford, MA, USA) and Evaporative Light Scattering Detector (Merck). The 1-DNJ standard 1.5 mg and HJ extract 100 mg were melted in 50% methanol and filtered through a 0.22 μm polyvinylidene fluoride membrane. For the high-performance liquid chromatography (HPLC) analysis, HPLC-grade distilled water (with 0.1% trifluoroacetic acid) (A) and acetonitrile (with 0.1% trifluoroacetic acid) (B) were used. The sample analysis time was 0–60 min. The solvent speed was maintained at 0.9 ml/min, with 10 μL of sample being injected.

### Cohort 1: *in vivo* study

#### Experimental groups.

**Fig 1:**
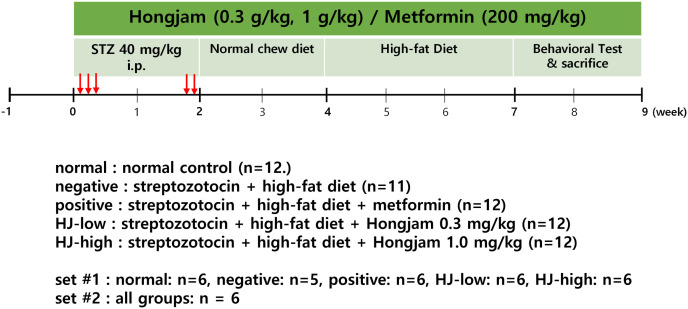
Schematic diagram of the experimental outline. Streptozotocin(STZ) was injected 5 times between the 1^st^ ~ 3^rd^ week. High fat diet was provided during the 4^th^ ~ 9^th^ week. Hongjam (HJ) was given intragastric and metformin i.p. injected during the whole experimental period. Behavioral measurements were performed between the 7^th^ ~ 9^th^ week. In set #1 animal numbers per group was 5 ~ 6. In set #2, animal numbers per group was 6.

This study was performed on 2 sets of animals. The first set was 7-week-old male C57Bl/6 mice were purchased from Saeron Bio. Inc (Kyunggi, Korea). Mice were housed 4–6 per cage in climate-controlled quarters (24 ± 1°C, 55 ± 5% relative humidity) maintained with a 12-h light and 12-h dark cycle. The animals were provided with standard rodent chow (AIN-93G) and water *ad libitum.* Mice were randomly divided into five experimental groups (G Power 3.1.9.4.; Conhen’s *f* = 0.40, Power 0.80, α = 0.05). 1) Normal: normal saline i.p. + regular diet (n = 6). 2) Negative: streptozotocin (S0130-50 mg, Sigma-Aldrich) 40 mg/kg i.p. + high-fat diet (n = 5). 3) Positive: streptozotocin 40 mg/kg i.p. + high-fat diet + metformin 200 mg/kg (metformin hydrochloride, PHR1084−500 mg, Sigma-Aldrich) (n = 6). 4) HJ-low: streptozotocin 40 mg/kg i.p + high-fat diet + HJ 0.3 mg/kg (n = 6). 5) HJ-high: streptozotocin 40 mg/kg i.p. + high-fat diet + HJ 1.0 mg/kg (n = 6). This set of mice were used for body weight, blood glucose, Morris water maze, open field test, Y-maze test, passive avoidance test, and western blot measurements. The second set of animals were an additional 6 mice each for groups, and was used for body weight, blood glucose and Morris water maze measurements. The formulation of regular diet and high-fat diet are summarized in Table 1. A schematic diagram of the experimental outline is provided in Fig 1.

**Table 1:** Dietary formula composition table for feeding.

Class	Ingredients	Normal diet AIN-93G (g/kg)	60% High fat diet (D12492; g/kg)
Protein	Casein, 30 Mesh	200	258.4
Protein	L-Cystein	3	3.9
Carbohydrate	corn starch	397.5	
Carbohydrate	Maltodextrin 10	132	161.5
Carbohydrate	Sucrose	100	88.9
Fiber	Cellulose	50	64.6
Fat	Soybean Oil	70	32.3
Fat	Lard	0	316.6
Mineral	Mineral Mix	35	12.9
Mineral	DiCalcium Phosphate	0	16.8
Mineral	Calcium Carbonate	0	7.1
Mineral	Potassium Citrate	0	21.3
Vitamin	Vitamin Mix	10	12.9
Vitamin	Choline Bitartrate	2.5	2.6
Antioxidant	tBHQ	0.01	0
Dye	FD&C Blue Dye #1	0	0.06
Sum		1000	1000

*tBHQ.

After a week of acclimatization to the facility, streptozotocin was intraperitoneally injected 5 times during the first 2 weeks (days 1,2,3, 13, and 14). After 2 weeks, mice were provided with a high-fat diet (D12492, Rodent Diet with 60 kcal% fat, Research Diet, U.S.; Saeronbio Inc.) for 3 weeks except the Normal group. Behavioral measurements were performed from the 7^th^ week, then animals were sacrificed at the 10^th^ week for molecular analysis. In the group ‘Positive’, metformin was given instead of HJ for the same duration. During the experimental period, body weight and blood glucose (glucometer; Acura view, Diasense Co. Seoul, Korea) were measured weekly.

#### Behavioral measurements.

Open field (OFT): The OFT was conducted in dimly illuminated conditions in a white opaque plexiglas chamber (50 × 50 × 40 cm). Mice were gently placed inside the chamber, and their movements were recorded for 30 min and analyzed using the Smart 3.0 video-tracking software (Panlab, Barcelona, Spain). The chamber device’s inside was cleaned with 70% ethanol and DW after each experiment.

Y-maze: Y-maze was performed to test working memory. Each arm was labeled—A, B, and C—and after putting the mouse in the center of the maze, the movement path of the mouse was recorded for 10 min. This test was based on the principle that mice prefer to explore novel places, which would result in them entering three different paths one by one. The movement path of the mouse was recorded and analyzed using the SMART 3.0 Software. Alternation triplet (%) is defined as alternation divided by (Total entries – 2) × 100.

Passive avoidance (PA): The PA test was performed to examine long-term avoidance memory. The apparatus is composed of an illuminated compartment (17 × 12 × 15 cm) with a light at the top and a dark compartment of the same size separated by a guillotine door (9 × 17 cm) that could be open for passing through. The floor was made of several stainless-steel grids (2.5 mm in diameter) and connected to an electric shock stimulator, which transfers electric current to the grid floor. The experiment progressed to two different trials, a training session for the acquisition of fear and a retention session for detecting whether the memory of fear was retained. Before the training session, each mouse was trained to adapt to the apparatus by freely moving through both chambers. In the training session, mice were initially put into the light compartment, facing away from the door. After 60 s, the guillotine door opened and as soon as all four paws of the mouse passed into the dark compartment, the guillotine door closed, and an electrical shock (0.55 mA, 2 s) was delivered. After 20 s, the mouse was removed from the apparatus and returned to the cage. The retention of fear memory was measured 24 h after the training session. Mice were placed in the light compartment for 60 s and as the guillotine door opened, the step-through latency time to enter the dark compartment was measured for 600 s (Hwang et al., 2017). If the mouse didn’t step into the dark compartment, the latency was recorded to be 600 s.

Morris water maze (MWM): The MWM test was performed to measure spatial learning and memory. A round metal tank 180 cm in diameter and 45 cm high was filled with tap water (22 ± 2 °C, depth 30 cm). A platform was submerged 0.5 cm under the water and was hidden from the animals by making the water nontransparent by adding white-colored paint. Four symbolic figures were attached to the wall in well-sighted places to the mice. The training lasted 5 days and was performed four times per day. During the four daily trials, the starting point differed on each trial. Training sessions continued until the mouse climbed up to find the platform, and after 1 min, if the platform was not found, the mouse was guided to the platform. The time to reach the platform was measured. On the 6th day, the probe test was performed without the platform. The trajectories of the swimming of the mice were tracked for 90 s and the time spent in the virtual quadrant where the platform was placed was measured. For measurement and analysis, SMART 3.0 was used.

#### Western blot.

Animals were deeply anesthetized and then rapidly sacrificed. Their dissected prefrontal cortex and hippocampus tissues were stored at −80°C until use. Tissues were homogenized with lysis buffer containing 1% each of phosphatase inhibitor cocktails 2 and 3 (P5726, P044, Sigma-Aldrich) in PRO-PREP™ (17081, iNtRON Biotechnology, Seongnam, South Korea) and centrifuged at 13,000 rpm for 10 min at 4°C. Protein concentrations were measured using DC™ Protein Assay Reagents A, B, and S (5000113, 5000114, 5000115, BIO-RAD, Hercules, CA, USA). After electrophoresis using 10% or 12% SDS-polyacrylamide gel, the gel phase proteins were transferred to a nitrocellulose membrane (162–0115, BIO-RAD). Membranes were incubated for 1 h at room temperature in 5% nonfat dry milk (170–6404, BIO-RAD) dissolved in TBS-T (0.1% tween 20 in Tris-buffered saline) for blocking. Primary antibodies were diluted in 5% nonfat dry milk in TBS-T and incubated overnight at 4°C. The next day, the membranes were washed thrice for 10 min with TBS-T. Secondary antibodies were diluted with 5% nonfat dry milk in TBS-T and incubated for 1 h at room temperature. After washing thrice for 10 min with TBS-T, luminescence was induced using a Pierce™ ECL Western Blotting Substrate Kit (32106, Thermo Fisher Scientific, Waltham, MA, USA), and the samples were photographed with an EZ-Capture MG (ATTO, Tokyo, Japan). Band density was analyzed using ImageJ software (NIH, Bethesda, MD, USA). The antibodies used in this experiment were: brain-derived neurotrophic factor (BDNF; PA5–85730, 1:1000, Invitrogen), synaptophysin (ab14692, 1:5000, Abcam), beta-actin (sc-47778, 1:5000, Santa Cruz), GAPDH (97166S, 1:3000, Cell signaling), anti-rabbit IgG (H + L) HRP conjugate (W4011, 1:1000, Promega, Madison, WI, USA), and anti-mouse IgG (H + L) HRP conjugate (W4021, 1:5000, Promega).

### Cohort 2: in vitro study

#### Materials.

DL-2-Amino-5-phosphonopentanoic acid (DL-AP5, 0105, TOCRIS, United Kingdom), 4-(2-hydroxyethyl)-1-piperazineethanesulfonic acid (HEPES, H4034), L-glutamine (G-8540), D-glucose (G7021), Sodium chloride (NaCl, S7653), potassium chloride (KCl, P5405), calcium chloride dihydrate (CaCl_2_, C7902), magnesium chloride hexahydrate (MgCl_2_, M2393), sodium carbonate (NaHCO_3_, S5761), and penicillin-streptomycin (P4333) were purchased from Sigma-Aldrich (St. Louis, MO, USA). Sodium dihydrogen phosphate (NaH_2_PO_4_, AB15ES) was obtained from DAEJUNG (Siheung, South Korea). The minimum essential medium (MEM, LM 007–01) and Hank’s balanced salt solution (HBSS, LB 003–01) were bought from Welgene (Kyungsan, South Korea). Donor horse serum and sterile filtered (HS, S0910-500) were obtained from Biowest (Nuaillé, France). All reagents for the whole experiment were research-grade products. All animal studies were conducted in accordance with the NIH Guide for the Care and Use of Laboratory Animals. The protocols were approved by the Institutional Animal Care and Use Committee of Kyung Hee University (KHUASP-21–068).

#### Organotypic hippocampal slice cultures (OHSCs).

The brain was quickly extracted from the male 7-day-old SD rats (from Saeron Bio. Inc., Kyunggi, Korea) after decapitation and immersed in a cold HBSS medium with 20 mM of HEPES immediately. Then the hippocampus extracted from the temporal lobe of the brain was cut to a thickness of 350 µm with a tissue chopper (Mickle Laboratory Engineering Co., Gomshall, UK). Each of the six-well plates filled with 1 mL of culture medium (MEM 50% v/v + HBSS 25% v/v + HS 25% v/v, supplemented with 20 mM HEPES, 5.25 g/L D-glucose, 1 mM L-glutamine, and 1% v/v penicillin-streptomycin; pH = 7.1) was added 0.4 µm membrane culture inserts (Mi8llicell-CM;). Approximately 5–6 tissue slices were placed on each insert. The culture medium was changed every two days, and the slices were incubated at 35°C with 5% CO_2_ for 12–14 days before the experiment.

#### Preparation of organotypic hippocampal tissue on the micro-electrode array (MEA) probes.

A single stabilized hippocampal slice tissue was delicately removed from a membrane of the insert with a soft brush and dropped into the artificial cerebrospinal fluid (aCSF; 114 mM NaCl, 3 mM KCl, 1.1 mM NaH_2_PO_4_, 2.5 mM NaHCO_3_, 25 mM D-glucose, 2 mM CaCl_2_, 1.3 mM MgCl_2_, and 20 mM HEPES; pH 7.4). The isolated hippocampal slice tissue was attached to an 8 × 8 MEA with 10 µm-diameter electrodes which were coated with 0.01% polyethyleneimine (PEI). MEA system consisted of a stimulator, amplifier, temperature control unit, and computer software for data acquisition. The slice tissue was stabilized in aCSF for 30 min at 33°C with 95% O2, and 5% CO2 gas aeration.

#### Induction of long-term potentiation (LTP) and treatment for hippocampal slice.

Bipolar electrical stimulation was applied to the stratum radiatum region of cornu ammonis (CA) 2 and CA3 to induce the Schaffer collateral and commissural pathways. The intensity of the bipolar stimulation was set to 160 μA, 240 ms per phase optimized to provide 40%–65% of the maximum tissue response. Theta burst stimulation (TBS) consisted of 300 biphasic pulses and three trains at 100 Hz for 1 s each at 5 min intervals. Each experiment to induce LTP was planned with a total 90 min protocol and consisted of 30 min of field excitatory postsynaptic potentials (fEPSPs) recordings, 10 min of TBS, and 50 min of fEPSP measurements after TBS. The Schaffer side and commissural pathways were selected by the morphological structure of the hippocampus tissue and the appropriate reaction by bipolar electrical stimulation. During the experiment, fresh aCSF was continuously injected into the hippocampal slice at a rate of 3 mL/min. AP5 (50 μM) and HJ (0.1, 1, 10 mg/ml) were treated with aCSF 10 min after the recording started.

All data were sampled from 60 recording channels at 25 kHz using Recorder-Rack software (Multi-Channel Systems). Experimental results data consisting of analog MEA signals with MC_Rack (v.3.2.1.0, Multi-Channel Systems, Reutlingen, Germany) were converted to digital form, of which EPSPs over 80 mV were selected using trigger mode. A custom MATLAB (v.7.0.1, Mathworks, Inc., MA, USA) program was used to remove stimulus artifacts and integrate the evoked field potential trajectory as reported previously.

### Statistical analysis

The significance of the difference in the mean of the data was analyzed by ANOVA, repeated-measure ANOVA using SPSS version 20 (IBM, Armonk, NY).

## Results

### Measurement of 1-DNJ in HJ extract

**Fig 2:**
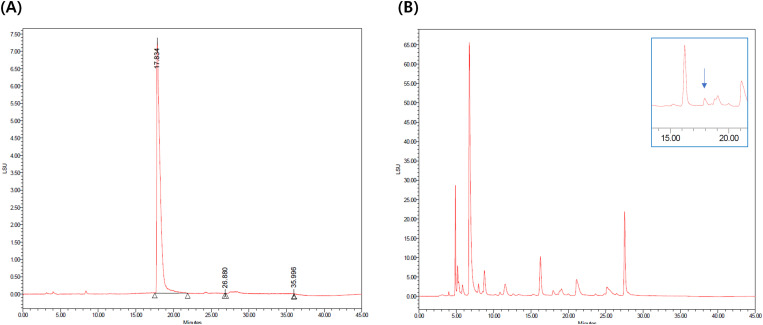
HPLC measurement of 1-Deoxynojirimycin (1-DNJ) in standard and HJ extract. (A) The standard of 1-DNJ peaks at 17.8 min (area 99.9%). (B) As a result of duplicate measurement, the 1-DNJ content of the HJ extract is 1.42 ± 0.01 mg/g.

The retention time of 1-DNJ standard peak was 17.834 min on the HPLC. The content of 1-DNJ in the HJ extract was 1.42 ± 0.01 mg/g (Fig 2). According the this measurement, the administered dose of 1-DNJ was in the HJ-low group was 0.426 μg/kg (HJ 0.3 mg/kg) and 1.42 μg/kg in the HJ-high (HJ 1 mg/kg) group. In hippocampal organic culture, doses of 0.1, 1, and 10 mg/mL of HJ extract corresponded to 1-DNJ of 0.142, 1.42, and 14.2 μg/mL.

### HJ doesn’t affect body weight but decreases the non-fasting blood glucose in streptozotocin + high-fat diet treated mice

**Fig 3:**
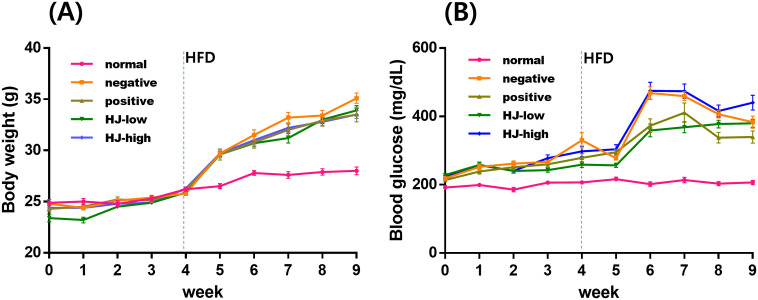
HJ does not reduce body weight but lowers blood glucose in a cognitive impairment mouse model. (A) Body weight. There was a change in body weight by time (within effect) [F (9, 486) = 756, p < 0.001], group (between effect) [F (4,54) = 16.7 p < 0.001] and within-between interaction [F (36, 486) = 20.1, p < 0.001]. According to post-hoc analysis, the negative group gained more than the normal group (p < 0.001). There was no difference among the negative, positive, HJ-low, and HJ-high groups. (B) Non fasting blood glucose. There was a change in blood glucose by time (within effect) [F (9, 486) = 177.5, p < 0.001], group (between effect) [F (4,54) = 56.9, p < 0.001, and within-between interaction [F (36, 486) = 14.1, p < 0.001]. According to post-hoc analysis, the negative group had higher glucose concentration than the normal mice (p < 0.001). Compared to the negative group, blood glucose concentration was lower in the positive group (p = 0.003) and the HJ-low group (p = 0.001), but not in the HJ-high group. All data points are mean ± SEM. HFD: high-fat diet. normal: normal saline i.p. + regular diet (n = 12). negative: streptozotocin 40 mg/kg i.p. + high-fat diet (n = 11). positive: streptozotocin 40 mg/kg i.p. + high-fat diet + metformin 200 mg/kg (n = 12). HJ-low: streptozotocin 40 mg/kg i.p + high-fat diet + HJ 0.3 mg/kg (n = 12). HJ-high: streptozotocin 40 mg/kg i.p. + high-fat diet + HJ 1.0 mg/kg (n = 12). Repeated Measures ANOVA, LSD post-hoc test.

The body weight and blood glucose concentrations were monitored throughout the experimental period (0–9^th^ week; Fig 3). As high-fat diet feeding started in the 4^th^ week, the body weight increased in the high-fat diet-fed groups (negative, positive, HJ-low, and HJ-high). However, there was no difference in the body weight among high-fat diet-fed groups. Non-fasting blood glucose concentration increased in all high-fat diet fed groups and was decreased in the positive and HJ-low groups compared to the negative group.

### HJ improves contextual memory in the Morris water maze

**Fig 4:**
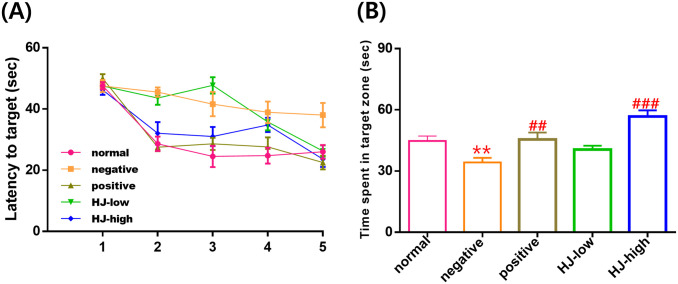
Effect of HJ on contextual memory. (A) Latency to the target in the Morris water maze. Effect by training days (within effect): F(4,216)=60.5, p < 0.001. Effect by group (between effect): F(4,54)=10.8, p < 0.001. Training time x group interaction: F(16,216)=5.0, p < 0.001. According to the post-hoc test, the latency to find the target increased in the negative group compared to the normal group (p < 0.001). Compared to the negative group, the latency reduced in the positive (p < 0.001), and HJ-high (p < 0.001) groups. (B) Time in target quadrant in the Morris water maze probe trial. Group effect: F(4,54)=9.37, p < 0.001. Post-hoc test: normal vs negative, p = 0.008, negative vs positive, p = 0.005, negative vs HJ-high, p < 0.001. All data points are mean ± SEM. normal: normal saline i.p. + regular diet (n = 12). negative: streptozotocin 40 mg/kg i.p. + high-fat diet (n = 11). positive: streptozotocin 40 mg/kg i.p. + high-fat diet + metformin 200 mg/kg (n = 12). HJ-low: streptozotocin 40 mg/kg i.p + high-fat diet + HJ 0.3 mg/kg (n = 12). HJ-high: streptozotocin 40 mg/kg i.p. + high-fat diet + HJ 1.0 mg/kg (n = 12). ** p < 0.01 vs normal, ^##^ p < 0.01, ^###^ p < 0.001 vs negative. Repeated measure ANOVA and one-way ANOVA, LSD post-hoc test.

After 3 weeks of a high-fat diet, contextual memory-related behavioral changes were measured with the MWM test (Fig 4). The negative group took longer to find the platform than the normal group (p < 0.001). Compared to the negative group, the latency to find the platform was less in the positive (p < 0.001), and HJ-high (p < 0.001) groups. In the probe test performed on the 6^th^ day, time spent in the target zone was reduced in the negative compared to the normal group (p = 0.008), and compared to the negative, the time increased in the positive (p = 0.005) and the HJ-high (p < 0.001) groups.

### HJ increases spontaneous locomotion and avoidance memory but not working memory

**Fig 5:**
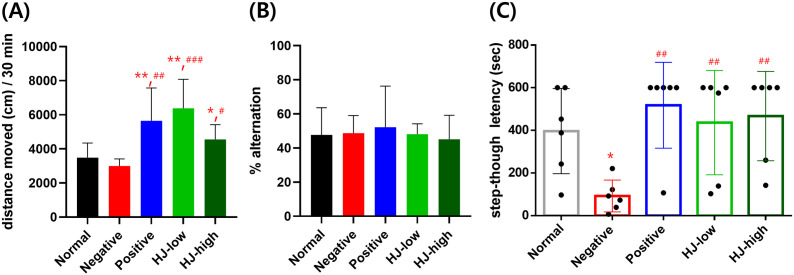
HJ increased spontaneous locomotion (A), didn’t affect working memory (B), and improved avoidance memory (C). (A) Distance moved in the open field test. Group effect F (4,25) = 7.3, p < 0.001. Post-hoc test: Normal vs. negative, p = 0.52, negative vs. positive, p = 0.001, negative vs. HJ-low, p < 0.001, negative vs. HJ-high, p = 0.046. (B) Percent alternation in the Y-maze test. Group effect F (4,25) = 0.098, p = 0.98. (C) Step-though latency in the passive avoidance test. Group effect: F (4,25) = 4.46, p = 0.007. Post-hoc test: normal vs. negative, p = 0.012, negative vs. positive, p = 0.001, negative vs. HJ-high, p = 0.005. negative vs. HJ-high, p = 0.003. All data points are mean ± SEM. * p < 0.05, ** p > 0.01 vs. normal, ^#^ p < 0.05, ^##^ p < 0.01, ^###^ p < 0.001 vs. negative. Repeated measure ANOVA and one-way ANOVA, LSD post-hoc test. HJ: Hongjam. Normal: normal saline i.p. + regular diet (n = 6). Negative: streptozotocin 40 mg/kg i.p. + high-fat diet (n = 6). Positive: streptozotocin 40 mg/kg i.p. + high-fat diet + metformin 200 mg/kg (n = 6). HJ-low: streptozotocin 40 mg/kg i.p + high-fat diet + HJ 0.3 mg/kg (n = 6). HJ-high: streptozotocin 40 mg/kg i.p. + high-fat diet + HJ 1.0 mg/kg (n = 6). ANOVA, Tukey’s HSD post-hoc test.

Also, spontaneous locomotion in the OFT, working memory in the Y-maze test, and avoidance memory in the PA test were measured (Fig 5). There was no difference between normal and negative groups but compared to the normal and negative groups, positive, HJ-low, and HJ-high groups showed more activity in the open field (Fig 5A). There was no difference in percent alternation in the Y-maze among all experimental groups (Fig 5B). Step-though latency measured 24 h after the electric shock, was reduced in the negative group, but metformin (positive) and HJ treatment prevented the shortening of latency (Fig 5C).

### HJ increases the hippocampal expression of BDNF but not synaptophysin

**Fig 6:**
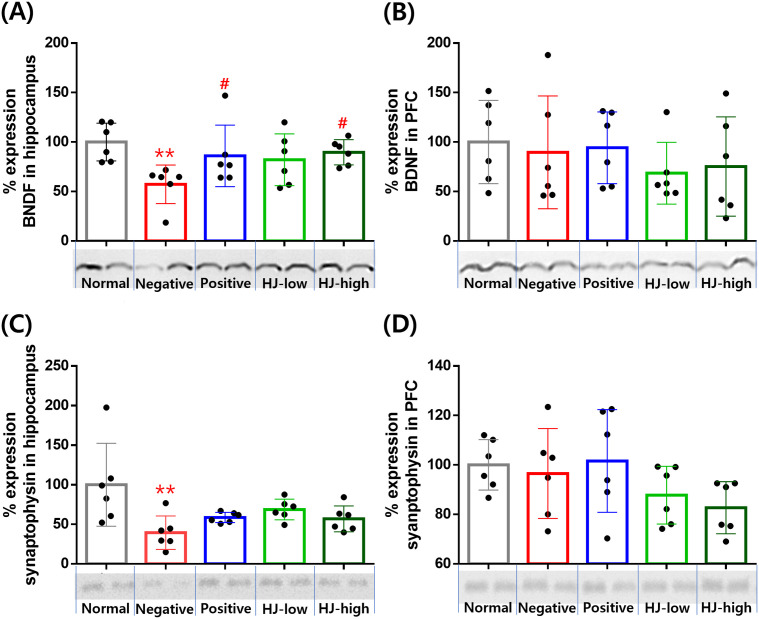
HJ increased the hippocampal expression of BDNF but didn’t increase the hippocampal expression of synaptophysin and prefrontal cortex expression of BDNF and synaptophysin. (A) BDNF expression in the hippocampus. Group effect F (4,25) = 2.9, p = 0.04. Post-hoc test: normal vs. negative, p = 0.003, negative vs. positive, p = 0.037, negative vs. HJ-low, p = 0.07, negative vs. HJ-high, p = 0.02. (B) BDNF expression in the prefrontal cortex (PFC). Group effect F.(4,25). = .0.53, p = 0.71. (C) Synaptophysin expression in the hippocampus. Group effect: F (4, 25) = 4.1, p = 0.011. Post-hoc test: normal vs. negative, p = 0.001, negative vs. positive, p = 0.23, negative vs. HJ-high, p = 0.07. negative vs. HJ-high, p = 0.28. All data points are mean ± SEM. ** p > 0.01 vs. normal, ^#^ p < 0.05 vs. negative. ANOVA, LSD post-hoc test. HJ: Hongjam. PFC: the prefrontal cortex. Normal: normal saline i.p. + regular diet (n = 6). Negative: streptozotocin 40 mg/kg i.p. + high-fat diet (n = 6). Positive: streptozotocin 40 mg/kg i.p. + high-fat diet + metformin 200 mg/kg (n = 6). HJ-low: streptozotocin 40 mg/kg i.p + high-fat diet + HJ 0.3 mg/kg (n = 6). HJ-high: streptozotocin 40 mg/kg i.p. + high-fat diet + HJ 1.0 mg/kg (n = 6). ANOVA, Tukey’s HSD post-hoc test.

After behavioral measurements, the hippocampus and prefrontal cortex were extracted for BDNF and synaptophysin expression measurement using western blot (Fig 6). In the hippocampus, BDNF expression was reduced in the negative group, and in the positive and HJ-high groups, BDNF expression was higher than that in the negative group (Fig 6A). Hippocampal synaptophysin expression was also reduced in the negative group, but there was no difference in positive, HJ-low, or HJ-high compared to the negative group (Fig 6C). In the prefrontal cortex, there was no difference in the expression of BDNF and synaptophysin (Figs 6B,D).

### HJ suppresses AP5-induced LTP blockade

**Fig 7:**
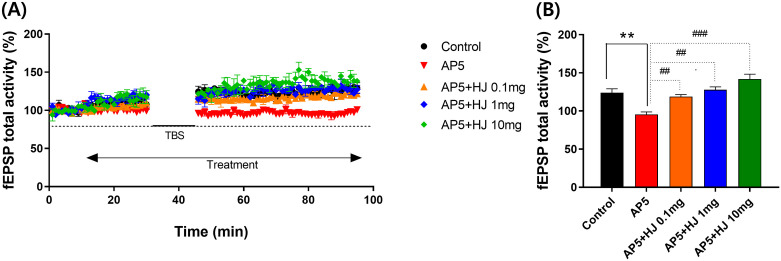
HJ improved hippocampal long-term potentiation suppressed by an NMDA antagonist. (A) fEPSP total activity (%) change induced by TBS while HJ application in organotypic cultured hippocampus. (B) Average of fEPSP total activity of 30–40 min after TBS during treatment of HJ (0.1, 1, and 10 mg/mL) in the organotypic cultured hippocampus. F (4, 10) = 22.535, *p *< 0.001. n = 3. All data points are mean ± SEM. ** p > 0.01 vs. control, ^##^ p < 0.01, ^###^ p < 0.001 vs. AP5. ANOVA, LSD post-hoc test. HJ: Hongjam. TBS: Theta burst stimulation. fEPSP: field excitatory postsynaptic potentials. AP5: DL-2-Amino-5-phosphonopentanoic acid. aCSF: artificial cerebral spinal fluid. Control: aCSF only. AP5: AP5 50 μM in aCSF. AP5 + HJ 0.1 mg: AP5 50 μM + HJ 0.1 mg/ml in aCSF. AP5 + HJ 1 mg: AP5 50 μM + HJ 1 mg/mL in aCSF. AP5 + HJ 10 mg: AP5 50 μM + HJ 10 mg/mL in aCSF. Repeated Measures ANOVA and ANOVA, Tukey’s HSD post-hoc test.

HJ was tested as if it could restore synaptic activity in the hippocampal CA1 induced by an NMDA antagonist, AP5. The time-dependent percentage change of fEPSP activity (Fig 7A) and the mean percentage change of fEPSP activity from 30–40 min after TBS (100 Hz, 1s, 3 trains, 5 min interval) were pooled for analysis (Fig 7B). AP5 (50 µM) treatment inhibited the post-TBS stimulated fEPSP compared to the non-treated group. HJ (0.1, 1, and 10 mg/mL) disinhibited the effect of AP5 on the post-TBS stimulated fEPSP activity.

## Discussion

In this study, we hypothesized that HJ could improve memory in a cognitively impaired animal model induced by streptozotocin and a high-fat diet. Thus, HJ did not reduce the body weight but reduced blood glucose. It improved spatial memory, avoidance memory, and potentiated hippocampal LTP suppressed by an NMDA antagonist. It also increased the expression of BDNF in the hippocampus. Conversely, there was no change in the working memory-related performance, and the expression of BDNF and synaptophysin in the prefrontal cortex was not changed by HJ. In the hippocampus, synaptophysin was decreased, but HJ did not increase the expression of synaptophysin. In addition, HJ and metformin increased spontaneous locomotor activity in the OFT.

HJ is a steamed and freeze-dried powder of mature silkworm which is rich in proteins, omega-3 fatty acids, and essential minerals [8]. Products of silkworms are known for antioxidant properties which have neuroprotection and cognitive improvement effects [19]. Neuroprotective effects were related to serotonin which is a component of silkworm pupae [20]. Silk proteins such as fibroin and sericin have antioxidant and anti-inflammatory properties [21]. In addition, bioactive peptides, choline-containing compounds, and lipid contents have shown beneficial effects on brain health by providing nutritional support, neurotransmitter regulation, and stimulating the production of neurotrophic factors [22].

Streptozotocin and high-fat diet-treated animal models are used to induce a combination of cognitive impairments, amyloid and Tau pathology, neuroinflammation, oxidative stress, impaired insulin signaling, and vascular changes, which are factors that have been implicated in the development of AD [23]. This model can provide insights into the links between metabolic dysfunction, cognitive impairments, and neurodegenerative processes. This model has shown impairments in many aspects of cognitive functions such as learning and memory, attention, problem-solving, spatial navigation, flexibility, novelty, and social recognition [5]. There was an increased production and decreased clearance of Aβ and was suggested that this model might trigger tau phosphorylation in the brain by increased oxidative stress and neuroinflammation [24]. The increase in microglia activation and proinflammatory cytokine in the brain suggests that there is neuroinflammation. In addition, metabolic abnormalities caused by a high-fat diet can increase oxidative stress and reduce the clearance of Aβ and tau proteins [25]. In particular, insulin plays an important role in maintaining the function of the nervous system [26]. In this model, streptozotocin was used to reduce the amount of insulin by damaging pancreatic beta cells, and a high-fat diet was used for inducing insulin resistance Therefore, a decrease in the nervous system plasticity and cognitive function due to an abnormality of insulin signaling in brain tissue occurred. Moreover, neuroinflammation, amyloid, and tau lesions, and a decrease in energy metabolism due to mitochondrial dysfunction occurred. In addition, reduction in cerebral blood flow, disruption of the blood-brain barrier, and vascular inflammation resulting in reduced blood flow in the brain all contributed to cognitive decline [5].

In the MWM, modeled rodents have shown increased latency to find the platform and spent less time in the target quadrant as well as the thigmotaxis pattern of swimming [27]. There are studies reporting reductions in BDNF levels in the brain, which is abundant in the hippocampus and is a key modulator of synaptic plasticity involved in the formation of new memories [28,29]. Impairment of BDNF signaling is suggested to be a cause of cognitive impairment in this model. A decrease in the expression of synaptophysin has also been reported in this model [30]. This is related to deterioration of synaptic function, such as deterioration of neurotransmitter secretion in the synaptic vesicle, or deterioration of synaptic plasticity function, and is a common change in neurodegenerative diseases. However, there have been no results of testing the effect of silkworm in this model so far.

It occurred in our results that the increase in body weight by high-fat diet and streptozotocin was not reduced by metformin and HJ. But the non-fasting blood glucose was reduced in this model. Many previous studies have reported the hypoglycemic effect of metformin [31,32]. In C57Bl/6 mice treated with 9 weeks of high-fat diet and two injections of 40 mg/kg streptozotocin, metformin 200 mg/kg for 10 days reduced the blood glucose concentration [33]. In addition, in C57Bl/6 mice treated with 4 weeks of high-fat diet and a single injection of 100 mg/kg streptozotocin, metformin 200 mg/kg mixed in water were given 24 weeks did not affect the body weight but reduced the blood glucose concentration [34]. However, not all study results showed that metformin reduced blood sugar levels in this model. In C57Bl/6 mice given intraperitoneal streptozotocin 90 mg/kg for days, intragastric infusion of metformin 100 mg/kg and 200 mg/kg did not reduce the body weight and blood glucose but improved spatial memory performance [35].

Regarding the hypoglycemic effect of HJ, previous research results showed that 50 mg/kg PO of 1-DNJ reduced the absorption of glucose in the intestines in streptozotocin-induced type 2 diabetes model mice, in association with suppressed expression of SGLT1, and GLUT2 [13]. When 1-DNJ 1 mg/kg and mulberry extract 100–200 mg/kg (corresponding to DNJ 1 mg/kg) was fed to SD rats for 4 weeks, AMPK expression and beta-oxidation increased [36]. The 1-DNJ content of HJ used in this study was 0.426 μg/kg (HJ-low) and 1.42 μg/kg (HJ-high), which was lower than previous reports, hence, blood sugar level reduced.

As a result of 5 days of training in the watermaze, spatial memory was formed in the experimental groups except the negative group through a decrease in the time to find the platform. Also, metformin and high dose HJ (in the HJ-high group) improved spatial learning in high-fat diet and streptozotocin treated mice. In the probe trial, memory retention related behavior reduced by high-fat diet + streptozotocin (in the negative group), but metformin and HJ 1 mg/kg treatment improved the memory retention compared to the negative group. As shown previously, silkworm pupae extract improved memory in scopolamine-treated mice in association with inhibition of acetylcholine esterase inhibition, choline acetyltransferase induction, and antioxidant and mitochondrial function-improving effects [15,36]. It also had a synergistic effect with Angelica gigas in improving spatial learning and memory in this model mouse [37]. BF-7, a bombyx mori extract component, treated for 3 weeks increased the K-WAIS IQ test score of 4 individuals in their 20s and increased blood flow in the parahippocampal gyrus and medial temporal area [38]. Similarly, HJ appeared to improve contextual memory in the high-fat diet + streptozotocin mice model.

In SAMP8 mice, 1-DNJ 40 and 160 mg/kg improved spatial memory measured in the Morris water maze and reduced Aβ, BACE1 and the expression of inflammatory cytokines in the hippocampus [39]. However, in our results, improvement in spatial memory was observed even though the content of 1-DNJ in HJ was lower, indicating that components of HJ other than 1-DNJ may also be effective in improving spatial memory. Memory consolidation in the hippocampus requires N-glycosylation, which 1-DNJ inhibits the N-linked oligosaccharide processing in the ER. Intrahippocampal infusion of 3.2 and 32 μg/μL 1-DNJ suppressed the consolidation and reconsolidation of fear conditioning memory [40]. Therefore, using an appropriate dose of 1-DNJ rather than a high dose is more effective in improving cognitive function.

Spontaneous locomotion increased in the OFT by metformin and HJ compared to the normal and negative groups of mice. It is suggested that metformin can increase locomotion by increasing energy utilization due to increased insulin sensitivity and glucose utilization [40]. However, it may vary depending on the mouse strain, metformin dose, administration period, etc., and results on the effect of metformin on spontaneous locomotion are inconsistent [41]. As HJ contains various nutrients, it may also increase energy utilization and increase locomotor activity. Additionally, the influence of increased locomotion on other behavioral tests should be considered. Mice with more activity can find the hidden platform faster. Also, active mice may pass earlier through the chamber in the PA test, which may be misinterpreted as increased or decreased memory, respectively. However, increased locomotion would have reduced step-through latency, but HJ increased the latency in the passive avoidance test. During the training period, the time taken to find the hidden platform in HJ-treated mice was reduced to a non-significant level (p = 0.051) in the MWM. Therefore, increased locomotor activity was not a confounding factor leading to misinterpretation as a cognitive improvement effect. The effect of HJ’s on contextual memory was significant in the PC1, which is a combined parameter of training and probe tests. Additionally, there was no effect of increased voluntary movement on the Y-maze.

The PA test is a method of measuring avoidance memory that combines two elements: contextual memory related to the place where the mouse received an electric shock and memory of the fear of the electric shock. In this cognitively impaired model, avoidance memory was reduced, and both metformin and HJ restored the avoidance memory. A few research results show that metformin strengthens avoidance memory, and in this regard, the improvement of glucose metabolism, the reduction of insulin resistance, and the protective effect of metformin on the hippocampal neurons may be related to the effect of this improvement [42,43]. Similarly, results have shown that HJ is neuroprotective and improves avoidance memory [36,44]. Many research results show that silkworm has anti-diabetic and insulin resistance-improving effects, and the improvement in avoidance memory in this study is expected to be the result of neuroprotection and improving insulin resistance [45–47].

At the molecular level, the expression of BDNF increased in the hippocampus, but only metformin and high-dose HJ increased the expression of BDNF in this model. This was consistent with the behavioral measurement of contextual memory component (=PC1) in the MWM, indicating that hippocampal BDNF is important for contextual memory performance in the MWM which is a consistent finding from many studies. Streptozotocin plus a high-fat diet reduced, and metformin given to this model improved the hippocampal BDNF expression [28,48]. There was no clear finding that silkworm pupae can change hippocampal BDNF expression, but silk fibroin enzyme hydrolysate was reported to increase the expression of BDNF in a scopolamine-induced cognitive deficit model [49]. In the prefrontal cortex, BDNF was reported to reduce in some previous studies using streptozotocin + high-fat diet model animals [29], but there was also a report that BDNF was not decreased [50]. As our data did not show any change of BDNF in the prefrontal cortex, the discrepancy in results may be due to differences in laboratory conditions. Meanwhile, 160 mg/kg 1-DNJ increased the expression of BDNF in the hippocampus of SAMP8 mice [39]. This dose was about 100 times higher than the amount of 1-DNJ contained in HJ in our study, so HJ may contain BDNF-inducing substances in addition to 1-DNJ.

Synaptophysin decreased in the hippocampus in the streptozotocin + high-fat diet group, and both metformin and HJ didn’t increase the expression of synaptophysin in these mice. In the PFC, there was no change in the expression of synaptophysin. Like our result, there is a study showing that a high-fat diet and streptozotocin decrease synaptophysin in the hippocampus, but not in the PFC [51]. There was also a study showing that melatonin increased the expression of hippocampal synaptophysin in this model [30]. Meanwhile, sericin increased synaptophysin in the hippocampus of learned helplessness and sleep-deprived mice [52,53]. However, considering that there has been no report that synaptophysin is increased by silkworm pupae extract as in this study, HJ does not seem to affect the expression of synaptophysin. Therefore, the improvement of cognitive function improvement based on behavioral measurement may be because of HJ on BDNF.

To examine the effects of HJ on memory-related synaptic activity, long-term potentiation was measured in cultured hippocampal sections. TBS-induced LTP was suppressed by an NMDA antagonist, and HJ increased the suppressed LTP in a concentration-dependent manner at the range of 0.1 to 10 mg/ml. Direct injection of BDNF into the hippocampus or treatments that increase the hippocampal expression of BDNF increased LTP [54,55]. Additionally, BDNF alleviated LTP inhibition caused by NMDA antagonists [56]. Considering that BDNF is one of the critical factors for LTP and that HJ increased BDNF expression in the hippocampus, the increase in LTP by HJ may be related to the increased expression of BDNF [57].

These findings show that Hongjam has the potential to improve cognitive function in the process of dementia progression caused by insulin resistance. However, after the mechanism by which insulin resistance causes dementia is more directly revealed, research into the role of Hongjam in this process needs to be conducted.

## Conclusion

In a streptozotocin and high-fat diet-treated mice model, HJ does not affect weight gain but lowers blood glucose, improves spatial memory and avoidance memory, which are symptoms related to cognitive function. BDNF expression and long-term potentiation increased in the hippocampus which may be related to cognitive improvement. These effects can be explained by 1-DNJ but was not possible to attribute to the effect of 1-DNJ alone. Many studies show that silkworm-derived substances can improve memory and prevent cognitive decline. This study suggests that HJ can improve symptoms of dementia caused by insulin resistance.

## Supporting information

S1 Fig Blot images of BDNF. (TIF)

S2 Fig Blot images of synaptophysin. (TIF)

S3 Data Raw data1. (XLSX)

S4 Data Raw data 2. Electrophysiology MEA results.
(XLSX)
