# Correction: Memory improving effect of silkworm larva on insulin resistance related cognitive impairment model

**DOI:** 10.1371/journal.pone.0336167

**Published:** 2025-11-04

**Authors:** Joohyun Hwang, Junhyuk Choi, Seung-Yun Cha, In-Seo Lee, Ji Hye Yoon, Duk Jin Jung, Chang Wook Lee, Seong Ryul Kim, Ji Hae Lee, Byeongyeob Jeon, Ji-Ho Park, Sungho Maeng, Hyunwoo Park

The affiliation for the ninth author is incorrect. Ji Hae Lee is not affiliated with #4 but with #3: Department of Agricultural Biology, National Institute of Agricultural Sciences, Rural Development Administration, Wanju, Republic of Korea.
