# Supplementary figures and images for: Memory improving effect of silkworm larva on insulin resistance related cognitive impairment model
Source: PLoS One. 2025 Jul 28;20(7):e0328847. doi: 10.1371/journal.pone.0328847 (PMC12303296; doi:10.1371/journal.pone.0328847)

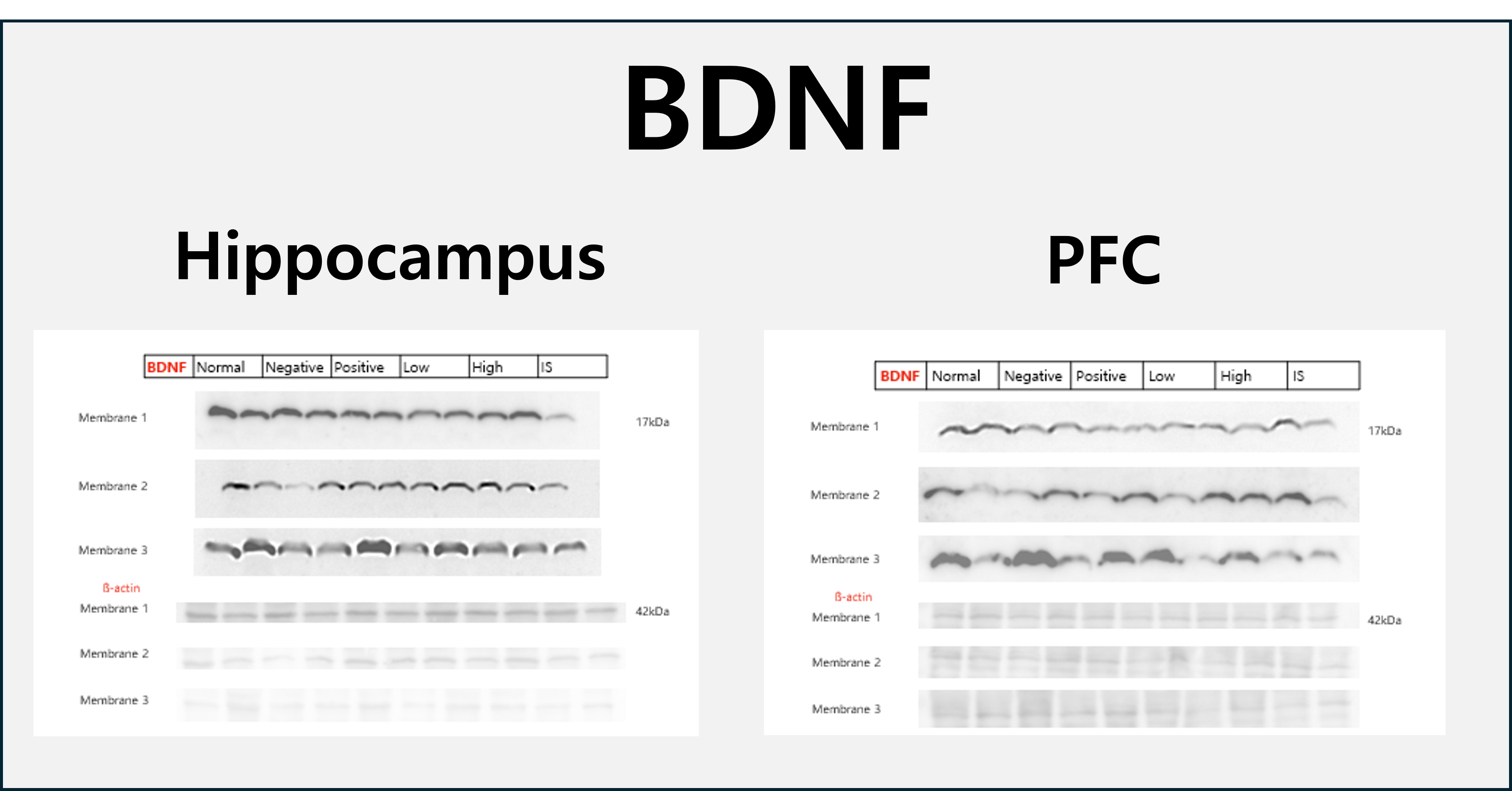

Supplement: S1 Fig — (TIF) [file pone.0328847.s001.tif]

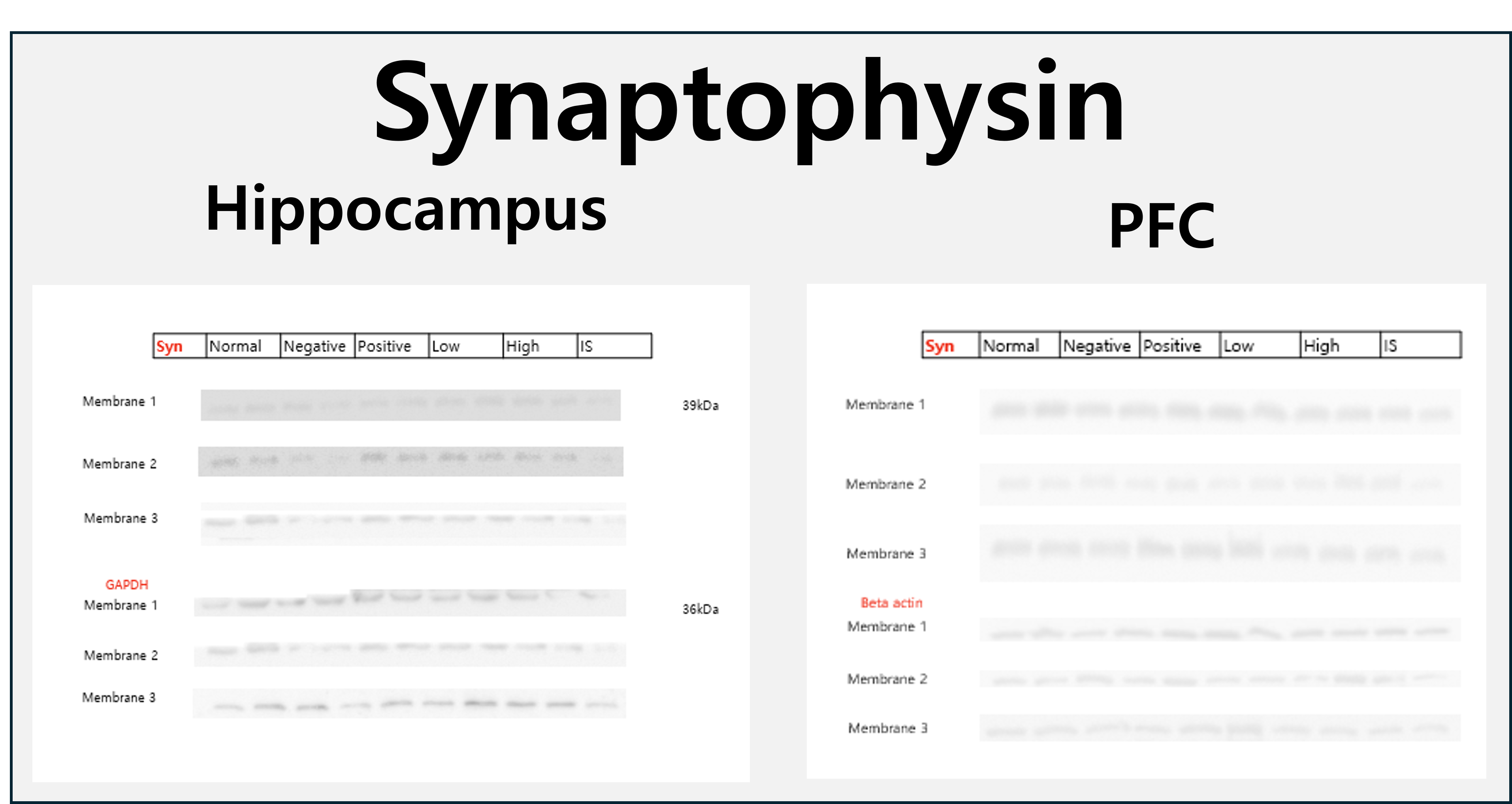

Supplement: S2 Fig — (TIF) [file pone.0328847.s002.tif]
